# Reviewed article: Azevedo SPF, Damião JJ, Castro IRR. Brazilian dietary guidelines: interfaces with the mother and child hospital context. Epidemiol Serv Saude. 2025:34;e20240391.

**DOI:** 10.1590/S2237-96222024v34e20240391.a

**Published:** 2025-05-02

**Authors:** Renata Paz Leal Pereira

**Affiliations:** University of Sao Paulo, Orthodontics and Peadiatric Dentistry

**Table:** 

Rating	Excellent	Good	Average	Below average	Poor
Interest		✓			
Quality					✓
Originality		✓			
Overall				✓	

**Table:** 

Questionnaire	Yes	No	Not applicable
Does the manuscript contain new and significant information to justify publication?		✓	
Does the Abstract (Summary) clearly and accurately describe the content of the article?		✓	
Is the problem significant and concisely stated?		✓	
Are the methods described comprehensively?		✓	
Are the interpretations and conclusions justified by the results?		✓	
Is adequate reference made to other work in the field?	✓		

## Manuscript Structure:

Length of article is: Adequate

Number of tables is: Adequate

Number of figures is: Adequate

Please state any conflict(s) of interest that you have in relation to the review of this paper (state “none” if this is not applicable): None

## Comments

O trabalho tem um tema relevante para promoção da saúde materno-infantil nutricional, mas estrutura do artigo está pobre de informações e organização. A introdução é construída em apenas dois parágrafos sem pontos importantes como conceito principal do tema do trabalho.

Há problemas graves de formatação. O texto precisa ser revisado para eliminar jargões em excesso, construções desnecessárias e vícios de linguagem. O texto apresenta resultados pouco completos e conclusivos, além de ter trechos que deveriam estar na discussão pois há opinião dos autores. Documentos importantes foram colocados em forma de link ao longo do texto e poderiam ser apêndices do trabalho. Há falta de referências em diversos trechos. O texto inteiro precisa ser revisado para aprimoramento tanto do conteúdo, quanto da formatação.

